# Prognostic impacts of repeated sepsis in intensive care unit on autoimmune disease patients: a retrospective cohort study

**DOI:** 10.1186/s12879-024-09072-y

**Published:** 2024-02-13

**Authors:** Jinming Yang, Jie Chen, Min Zhang, Qingsa Zhou, Bing Yan

**Affiliations:** 1 Department of Rheumatology and Immunology, West China Hospital, Sichuan University, No.37 Guoxue Alley, 610041 Chengdu, China; 2 Department of Rheumatology, People’s Hospital of Leshan, Leshan, China

**Keywords:** Autoimmune disease, Repeated sepsis, Critical care, Prognosis

## Abstract

**Background:**

Autoimmune diseases (ADs) may be complicated by sepsis when intensive care unit (ICU) admission. But repeated sepsis among AD patients has not been studied yet. The aim of this study is to investigate the impact of repeated in-ICU sepsis on the 1-year overall-cause mortality, septic shock and in-ICU death of AD patients.

**Methods:**

Data of AD patients with sepsis retrieved from Medical Information Mart for Intensive Care IV (MIMIC-IV) database were divided into the single group and the repeated group according to the frequency of in-ICU sepsis. Propensity score matching was used to balance inter-group bias. Cox proportional hazard regression and sensitivity analysis were utilized to assess the variables on mortality.

**Results:**

The incidence of repeated in-ICU sepsis in baseline was 19.8%. The repeated in-ICU sepsis was a risk factor for 1-year overall-cause mortality among AD patients (adjusted hazard ratio [HR] = 1.50, 95% CI: 1.16–1.93, *P* = 0.002), with robust adjusted HRs by the adjustment for confounders in the sensitivity analysis (all *P* < 0.01). Maximum Sequential Organ Failure Assessment (Max SOFA), Charlson comorbidity index (CCI) and Simplified Acute Physiology Score-II (SAPS-II) were risk factors for 1-year overall-cause mortality among AD with repeated sepsis (Max SOFA: HR = 1.09, *P* = 0.002; CCI: HR = 1.08, *P* = 0.039; SAPS-II: HR = 1.03, *P* < 0.001).

**Conclusions:**

Compared to single hit, repeated in-ICU sepsis was independently related to a higher risk of 1-year overall-cause mortality among AD patients. Assessment tools (Higher SOFA, CCI and SAPS-II scores) were closely linked to poor prognosis of AD with repeated sepsis and helped to reflect ill physical conditions for the patients.

**Supplementary Information:**

The online version contains supplementary material available at 10.1186/s12879-024-09072-y.

## Introduction

Autoimmune diseases (ADs) encompass a heterogeneous group of disorders characterized by the self-attack on the tissues or organs of patients themselves. The dysregulation of the immune system in ADs may lead to chronic inflammation, multiple organ damage, and various comorbidities, rendering the patients more susceptible to infections in the long-term periods [1].

Sepsis is another disorder that is attributable to a dysregulated immune response to an infection and successively progress to life-threatening multiorgan failure even deaths [2]. In the USA, severe sepsis is reported to occupy about 10% of all admissions to intensive care units (ICU) and its mortality highly reaches 30% [3]. Moreover, the coexistence between AD and sepsis is common in hospitalization, and the mortality of sepsis overlapping with ADs in ICU is up to 40% [1, 4, 5]. Owing to the high prevalence and high mortality of ADs with sepsis, improving health outcomes among this population has become a notable research focus.

Sepsis is currently found to be a process that starts with inflammation and transitions to a state of immunosuppression in the long period, and the latter phase predisposes individuals to secondary infection or repeated sepsis [6–8]. For infection-susceptible AD patients, the occurrence of sepsis may exacerbate the immunity dysregulation and cause recurrent sepsis episodes afterwards. To date, previous studies have demonstrated that recurrent sepsis was associated with higher mid- or long-term mortality for septic patients [9, 10]. However, the finding cannot be extrapolated directly to AD patients in ICU. As for AD population complicated with sepsis, it still remains poorly understood about the impact of single or multiple sepsis episodes on their long-term survival.

Therefore, our study extracted data of AD patients with different episodes of in-ICU sepsis from Medical Information Mart for Intensive Care IV (MIMIC-IV) database, and aimed to investigate the influence of repeated in-ICU sepsis on the prognosis of AD patients, with 1-year overall-cause mortality, septic shock and in-ICU death as outcomes.

## Materials and methods

### Data source

The data were extracted from MIMIC-IV database version 2.0 from 2008 to 2019 [11]. It is a publicly shared and widely-used database providing critical care information of tens of thousands of ICU patients at the Beth Israel Deaconess Medical Center. The acquisition of the data was permitted by the database and did not require ethical approval.

### Patient population and variables

**Fig. 1:**
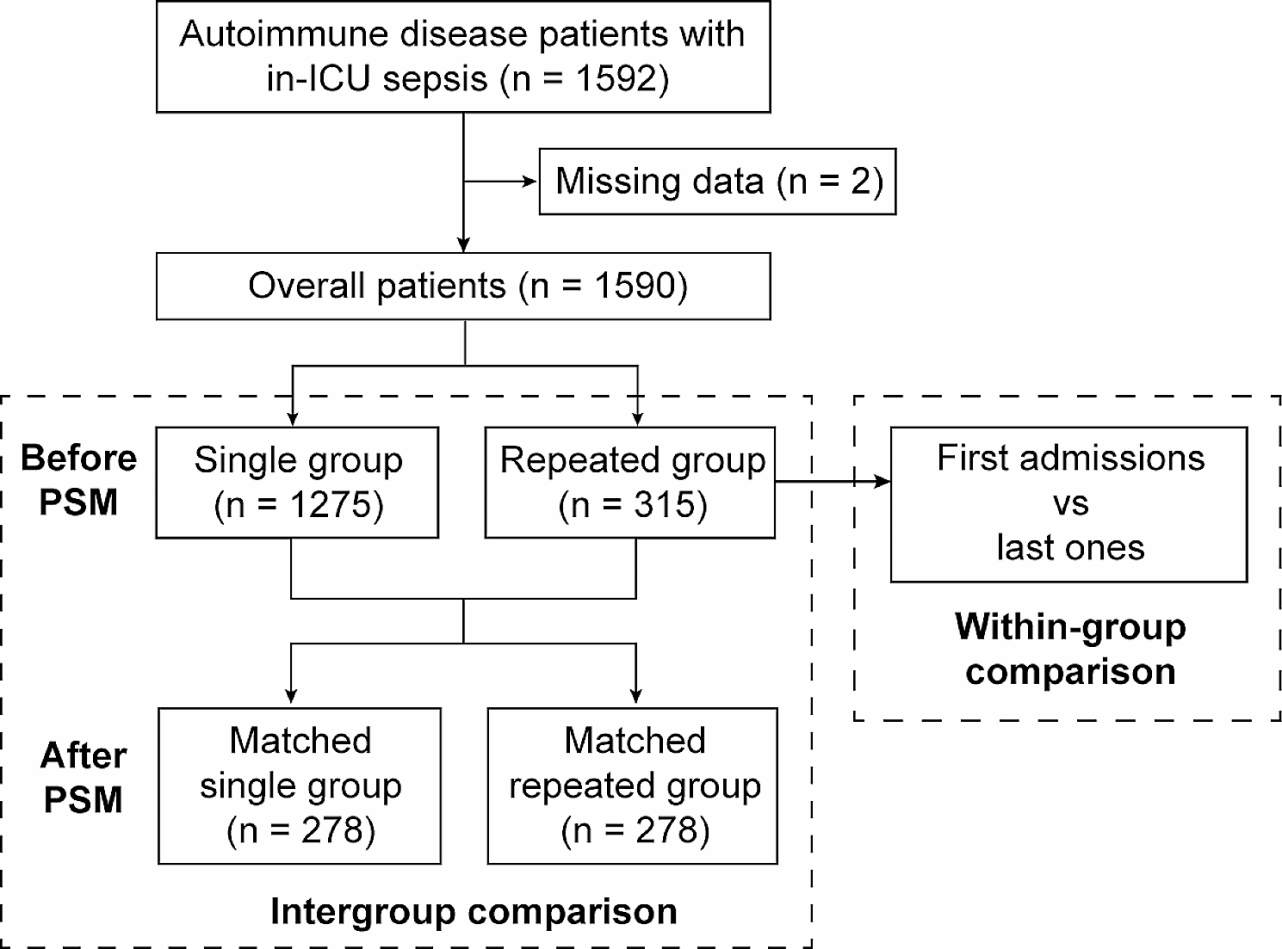
Flow chart of the patient selection and grouping. Abbreviations: PSM: propensity score matching

We selected patients over 18 years old who were admitted to the ICU with diagnosis of ADs, which encompassed systemic lupus erythematosus (SLE), rheumatoid arthritis, systemic sclerosis, psoriasis, ankylosing spondylitis, vasculitis, idiopathic inflammatory myopathies, autoimmune hepatic diseases, and inflammatory bowel diseases, namely Crohn’s disease and ulcerative colitis (Supplementary Table S1). Therein, AD patients diagnosed with in-ICU sepsis were retrieved. In-ICU sepsis was defined as a condition existing during the ICU stay and accorded with the Sepsis-3 criteria: an associated suspicion of an infection event and a Sequential Organ Failure Assessment (SOFA) score ≥ 2. We counted the frequency of in-ICU sepsis among these selected AD patients, and those ICU admissions when sepsis did not occur were excluded from the count. Patients with a single record of in-ICU sepsis were grouped into “single group”, before which they each did not experience a sepsis-related hospitalization. Those with records more than one time were into “repeated group”. We selected the last recodes of in-ICU sepsis among the repeated group to compare with the unique episode of the single group, namely the intergroup comparison. Within the repeated group, a within-group comparison of characteristics between the first admissions and the last ones was conducted (Fig. 1).

The variables assessed in the study were as follows: in-ICU sepsis frequency (single and repeated), age at admission (< 65 and ≥ 65 years old), gender, race (white, black, Hispanic, Asian, other and unknown), overlapping syndrome, length of ICU stay, comorbidities, maximum SOFA (Max SOFA), Charlson comorbidity index (CCI), Simplified Acute Physiology Score-II (SAPS-II), maximum of white blood cell (WBC) count, minimum of hemoglobin (Hb), minimum of platelet (PLT) count, use of glucocorticoids, immunosuppressants/biologics and intravenous immunoglobulin (IVIG) (Supplementary Table S2). The comorbidities embodied acute kidney injury, chronic kidney disease, atrial fibrillation, essential hypertension, heart failure, myocardial infarction, respiratory failure, chronic obstructive pulmonary disease (COPD), diabetes mellitus, human immunodeficiency virus (HIV) infection and malignancy. Overlapping syndrome patients were defined as those with the combination of more than one autoimmune diseases, or they would be marked with “no”. Those with unknown or no evidence of comorbidities and drug therapies were also labeled with “no”. SOFA score was a sum of quantified assessment of respiration, coagulation, central nervous system, renal system, cardiovascular system and liver function [12]. CCI was a measurement of the comorbidities based on ICD-9/ICD-10 codes [13] and the SPAS-II is used to assess the illness severity during the first 24 hours of each ICU stay [14]. Individuals with missed data of evaluation tools (Max SOFA, CCI and SAPS-II) or laboratory results were excluded.

### Primary and secondary outcomes

In view of the long-term immune dysregulation shared by ADs and sepsis as well as the commonly-used indexes in other previous studies [9, 15], we chose 1-year overall-cause mortality as primary outcome in this study. The occurrence of septic shock (ICD-code 78,552 and R6521) and in-ICU death were identified as the secondary outcomes, as they were commonly adverse events among AD patients in ICU [15–17]. Thereinto, the septic shock was the complication during ICU stay. The follow-up of patients started on the date of selected records of ICU admissions and lasted for one year. 1-year overall-cause mortality reflected deaths within a 1-year observation window, including in-hospital deaths and deaths after hospital discharge. Observations of fully one year were deemed as censored events.

### Statistical analysis

Considering the small number of missing data, we opted to address missing data by employing a deletion approach (Fig. 1). For the post-deletion data, Chi-square test was implied to assess the categorical variables in the baseline characteristics. If numeric variables of the two groups did not meet normal distribution through Shapiro-Wilk normality test (*P* < 0.05), they were shown as medians with inter-quartile ranges (IQRs) and their differences were evaluated by Wilcoxon rank sum test. To decrease the potential imbalance between the single group and the repeated group, a 1:1 propensity score matching (PSM) was applied. The algorithm principle of PSM was to match potential confounding variables based on logistic regression and nearest neighbor method with a caliper width of 0.02. All the variables we chose were put into PSM and the balances were acceptable if *P* > 0.05.

After PSM, the survival probability among these two groups was shown by Kaplan-Meier survival curve, and the comparison of survival was performed through Log-rank test. Further, univariate and multivariate Cox proportional hazard regressions were used to assess the independent risk factors on survival among AD patients with sepsis. If variables met *P* ≤ 0.05 in univariate analysis, they would be selected into the multivariate analysis. The proportional hazards assumption was examined by Schoenfeld residuals and was satisfied. To investigate whether the effect of in-ICU sepsis frequency on survival was influenced by other variables, sensitivity analysis was conducted where variables were included through forward selection in multivariate Cox proportional hazard regressions. Multivariate Cox regression analyses were further applied in the subgroup analysis of the single group and the repeated group. Moreover, comparisons of secondary outcomes between the two groups were evaluated by logistic regressions and adjusted by other variables.

All statistical analyses and visualization of results were performed using R version 4.0.5. A two-tailed *P* ≤ 0.05 was considered statistically significance.

## Results

### General description among AD patients

Before PSM, a total of 1590 AD patients with sepsis were selected, of whom 315 (19.8%) belonged to the repeated group (Table 1). Supplementary Table S1 provided further details on the distribution of specific AD diagnoses, among whom overlapping syndrome was observed in 90 patients (5.7%) (Table 1). Compared to the single group, the repeated group exhibited fewer white, longer length of ICU stays, higher scores for SOFA, CCI, and SAPS-II, lower levels of Hb and PLT, and a lower incidence of comorbidities including acute kidney injury, chronic kidney disease, essential hypertension, heart failure, respiratory failure and diabetes mellitus (all *P* < 0.05). However, this imbalance was eliminated after 1:1 PSM and there were 278 patients equally in each matched group (Table 1).

**Table 1:** The demographic and clinical characteristics of AD patients before and after PSM

Variables ^a^	Before PSM				After PSM		
	Single (*n* = 1275)	Repeated (*n* = 315)	*P* value	Single (*n* = 278)		Repeated (*n* = 278)	*P* value
Age at admission							
< 65 years old	499 (39.1%)	137 (43.5%)	0.177	104 (37.4%)		119 (42.8%)	0.226
≥ 65 years old	776 (60.9%)	178 (56.5%)		174 (62.6%)		159 (57.2%)	
Gender							
Male	578 (45.3%)	128 (40.6%)	0.150	109 (39.2%)		114 (41.0%)	0.729
Female	697 (54.7%)	187 (59.4%)		169 (60.8%)		164 (59.0%)	
Race							
White	927 (72.7%)	219 (69.5%)	0.003	194 (69.8%)		196 (70.5%)	0.505
Black	105 (8.2%)	46 (14.6%)		40 (14.4%)		34 (12.2%)	
Hispanic	31 (2.4%)	9 (2.9%)		3 (1.1%)		9 (3.2%)	
Asian	23 (1.8%)	9 (2.9%)		6 (2.2%)		8 (2.9%)	
Other	59 (4.6%)	14 (4.4%)		17 (6.1%)		13 (4.7)	
Unknown	130 (10.2%)	18 (5.7%)		18 (6.5%)		18 (6.5%)	
Overlapping syndrome	66 (5.2%)	24 (7.6%)	0.123	15 (5.4%)		20 (7.2%)	0.485
Length of ICU stay, days	3 (1–6)	3 (2–7)	0.032	3 (1–7)		3 (1–6)	0.674
**Comorbidities**							
Acute kidney injury	516 (40.5%)	163 (51.7%)	< 0.001	139 (50.0%)		135 (48.6%)	0.799
Chronic kidney disease	285 (22.4%)	119 (37.8%)	< 0.001	96 (34.5%)		91 (32.7%)	0.720
Atrial fibrillation	393 (30.8%)	103 (32.7%)	0.565	93 (33.5%)		85 (30.6%)	0.525
Essential hypertension	546 (42.8%)	92 (29.2%)	< 0.001	95 (34.2%)		88 (31.7%)	0.588
Heart failure	347 (27.2%)	139 (44.1%)	< 0.001	113 (40.6%)		112 (40.3%)	1.000
Myocardial infarction	206 (16.2%)	57 (18.1%)	0.457	57 (20.5%)		49 (17.6%)	0.450
Respiratory failure ^b^	438 (34.4%)	142 (45.1%)	0.001	119 (42.8%)		114 (41.0%)	0.731
COPD	92 (7.2%)	29 (9.2%)	0.283	19 (6.8%)		24 (8.6%)	0.525
Diabetes mellitus	334 (26.2%)	122 (38.7%)	< 0.001	101 (36.3%)		96 (34.5%)	0.723
HIV infection	11 (0.9%)	3 (1.0%)	1.000	5 (1.8%)		3 (1.1%)	0.722
Malignancy	132 (10.4%)	25 (7.9%)	0.237	17 (6.1%)		23 (8.3%)	0.412
**Quantified assessment tools**							
Max SOFA	6 (4–9)	6 (4–10)	0.009	6 (4–10)		6 (4–10)	0.679
CCI	6 (4–8)	7 (5–9)	< 0.001	7 (5–9)		7 (5–8)	0.553
SAPS-II	38 (29–48)	39 (31–48)	0.046	38 (29–49)		39 (30–47)	0.886
**Laboratory results**							
Max WBC count, K/𝜇L	16.00 (11.70–22.20)	16.00 (10.70–23.00)	0.907	16.05 (11.62–20.60)		15.75 (10.43–22.90)	0.969
Min Hb, g/dL	8.20 (7.10–9.55)	7.20 (6.60–8.65)	< 0.001	7.70 (6.73–8.88)		7.35 (6.70–8.80)	0.571
Min PLT, K/𝜇L	129.00 (80.50–192.00)	119.00 (59.50-179.50)	0.011	118.50 (69.25-196.75)		121.50 (60.00-186.75)	0.556
**Drug therapies**							
Glucocorticoids	95 (7.5%)	32 (10.2%)	0.141	23 (8.3%)		23 (8.3%)	1.000
Immunosuppressants/biologics	94 (7.4%)	47 (14.9%)	< 0.001	32 (11.5%)		37 (13.3%)	0.607
IVIG	29 (2.3%)	14 (4.4%)	0.053	6 (2.2%)		11 (4.0%)	0.324

^a^ Data was displayed as mean (SD), n (%), or median (IQR) unless otherwise stated

^b^ Respiratory failure consisted of acute respiratory failure, chronic respiratory failure and respiratory failure (unspecified), among which acute ones occupied 90.3% (≈ 533/580) before PSM, and the remaining two accounted for a minority of cases, totaling 9.7%

Abbreviations: AD: autoimmune disease; CCI: Charlson comorbidity index; COPD: chronic obstructive pulmonary disease; Hb: hemoglobin; HIV: human immunodeficiency virus; ICU: intensive care unit; IVIG: intravenous immunoglobulin; PLT: platelet; PSM: propensity score matching; SAPS-II: Simplified Acute Physiology Score-II; SOFA: Sequential Organ Failure Assessment; WBC: white blood cell

Within the unmatched repeated group (Supplementary Table S3), Max SOFA, CCI and SAPS-II significantly increased in the last admissions than those in the first admissions (all *P* < 0.05). But insignificant differences were presented in the comparison of comorbidities, laboratory examinations and drug therapies between the last and the first admissions.

### In-ICU sepsis on 1-year overall-cause mortality in survival analysis

**Fig. 2:**
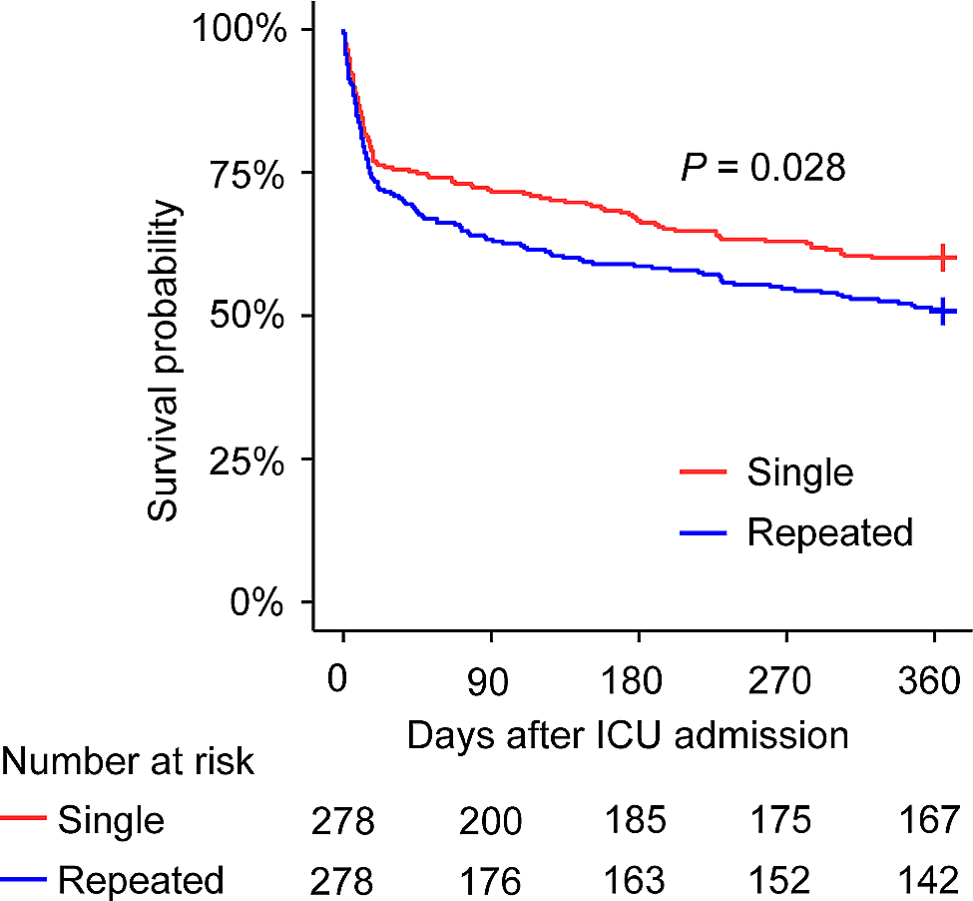
1-year overall-cause mortality among autoimmune disease patients with sepsis

In survival analysis where in-ICU sepsis frequency acted as a dichotomous variable, 1-year overall-cause mortality significantly ascended among patients in the repeated group than those in the single group (*P* = 0.028). Since the deaths of each group were fewer than 50%, median survival was unavailable. At the terminal of one year, the survival probability of the repeated group reached 50.7% and the single group reached 60.1% (Fig. 2).

### Risk factors for 1-year overall-cause mortality in univariate and multivariate analyses

The results of univariate and multivariate Cox proportional hazard regressions were shown in Table 2. Repeated in-ICU sepsis was a risk factor for the 1-year overall-cause mortality of AD patients with statistical significance (unadjusted hazard ratio [HR] = 1.32, 95% CI: 1.03–1.70, *P* = 0.028; adjusted HR = 1.50, 95% CI: 1.16–1.93, *P* = 0.002). Besides, respiratory failure, Max SOFA, CCI and SAPS-II were also risk factors for AD patients with sepsis (all *P* < 0.05). For three quantified comprehensive tools (Max SOFA, CCI and SAPS-II), an increase of 1 point was associated with a 2-13% increase in the risk of deaths (Max SOFA: adjusted HR = 1.13, 95% CI: 1.08–1.18, *P* < 0.001; CCI: adjusted HR = 1.09, 95% CI: 1.02–1.17, *P* = 0.016; SAPS-II: adjusted HR = 1.02, 95% CI: 1.01–1.03, *P* < 0.001). Length of ICU stay, working as a continuous variable, was associated with a 3% decrease in the risk of death (adjusted HR = 0.97, 95% CI: 0.95-1.00, *P* = 0.030). The use of immunosuppressants/biologics was a predictor for better prognosis (adjusted HR = 0.55, 95% CI: 0.35–0.87, *P* = 0.011).

**Table 2:** Univariate cox and multivariate Cox proportional hazard regressions of 1-year overall-cause mortality among overall AD patients

Variables	Univariate Cox			Multivariate Cox	
	Unadjusted HR (95% CI)	*P* value		Adjusted HR (95% CI)	*P* value
In-ICU sepsis frequency					
Single	Reference			Reference	
Repeated	1.32 (1.03–1.70)	0.028		1.50 (1.16–1.93)	0.002
Age at admission, years					
< 65 years old	Reference			Reference	
≥ 65 years old	1.79 (1.36–2.35)	< 0.001		1.27 (0.91–1.76)	0.161
Gender					
Male	Reference				
Female	1.15 (0.89–1.49)	0.281			
Race					
White	Reference				
Black	1.17 (0.81–1.67)	0.398			
Hispanic	1.55 (0.73–3.31)	0.254			
Asian	0.60 (0.22–1.63)	0.320			
Other	1.19 (0.70–2.03)	0.509			
Unknown	1.26 (0.78–2.06)	0.344			
Overlapping syndrome ^a^	0.91 (0.56–1.49)	0.709			
Length of ICU stay ^b^	1.03 (1.02–1.05)	< 0.001		0.97 (0.95-1.00)	0.030
**Comorbidities** ^**a**^					
Acute kidney injury	1.72 (1.33–2.21)	< 0.001		0.95 (0.71–1.27)	0.734
Chronic kidney disease	1.36 (1.05–1.75)	0.019		0.82 (0.60–1.12)	0.212
Atrial fibrillation	1.40 (1.09–1.81)	0.010		1.05 (0.79–1.39)	0.756
Essential hypertension	0.88 (0.67–1.15)	0.358			
Myocardial infarction	1.31 (0.97–1.77)	0.076			
Heart failure	1.51 (1.18–1.94)	0.001		0.94 (0.70–1.26)	0.675
Respiratory failure	1.97 (1.53–2.53)	< 0.001		1.46 (1.10–1.95)	0.009
COPD	1.59 (1.05–2.41)	0.028		1.13 (0.72–1.75)	0.601
Diabetes mellitus	1.05 (0.81–1.36)	0.727			
HIV infection	0.86 (0.28–2.70)	0.802			
Malignancy	1.92 (1.28–2.89)	0.002		1.23 (0.75–2.03)	0.417
**Quantified assessment tools** ^**b**^					
Max SOFA	1.14 (1.11–1.17)	< 0.001		1.13 (1.08–1.18)	< 0.001
CCI	1.16 (1.11–1.21)	< 0.001		1.09 (1.02–1.17)	0.016
SAPS-II	1.05 (1.04–1.06)	< 0.001		1.02 (1.01–1.03)	< 0.001
**Laboratory results** ^**b**^					
Max WBC count, K/𝜇L	1.00 (1.00-1.01)	0.087			
Min Hb, g/dL	0.90 (0.83–0.98)	0.014		1.01 (0.93–1.10)	0.792
Min PLT, K/𝜇L	1.00 (1.00–1.00)	0.123			
**Drug therapies** ^**a**^					
Glucocorticoids	0.80 (0.49–1.31)	0.374			
Immunosuppressants/biologics	0.60 (0.39–0.93)	0.023		0.55 (0.35–0.87)	0.011
IVIG	1.61 (0.85–3.03)	0.141			

^a^ The overlapping syndrome, comorbidities and drug therapies were included in Cox proportional hazard regressions, with unknown or no evidence of these variables set as the references (reference = “no”, described in **Materials and methods**)

^b^ Length of ICU stay, Max SOFA, CCI, SAPS-II, Max WBC count, Min Hb and Min PLT were included in Cox proportional hazard regressions as continuous variables

Abbreviations: AD: autoimmune disease; CCI: Charlson comorbidity index; CI: confidence interval; COPD: chronic obstructive pulmonary disease; Hb: hemoglobin; HIV: human immunodeficiency virus; HR: hazard ratio; ICU: intensive care unit; IVIG: intravenous immunoglobulin; PLT: platelet; SAPS-II: Simplified Acute Physiology Score-II; SOFA: Sequential Organ Failure Assessment; WBC: white blood cell

To explore whether in-ICU sepsis frequency was an independent risk factor from other covariates (respiratory failure, Max SOFA, CCI, SAPS-II, length of ICU stay and use of immunosuppressants/biologics), the sensitivity analysis showed relatively robust adjusted HRs of in-ICU sepsis frequency in Model 1–3 (all *P* < 0.01) (Supplementary Table S4).

### Max SOFA, CCI and SAPS-II on 1-year overall-cause mortality in subgroup analysis

We further conducted a subgroup analysis to investigate the effect of Max SOFA, CCI and SAPS-II on the mortality respectively in the single group and the repeated. As depicted in Table 3, the subgroup analysis revealed that Max SOFA, CCI, and SAPS-II were identified as risk factors for 1-year overall-cause mortality within both the repeated group (Max SOFA: HR = 1.09, 95% CI: 1.03–1.16, *P* = 0.002; CCI: HR = 1.08, 95% CI: 1.00-1.15, *P* = 0.039; SAPS-II: HR = 1.03, 95% CI: 1.01–1.04, *P* < 0.001) and the single group (Max SOFA: HR = 1.12, 95% CI: 1.05–1.20, *P* < 0.001; CCI: HR = 1.13, 95% CI: 1.04–1.21, *P* = 0.002; SAPS-II: HR = 1.02, 95% CI: 1.00-1.04, *P* = 0.038).

**Table 3:** Subgroup analysis of quantified assessment tool on 1-year overall-cause mortality among the single and the repeated group

Variables ^a^	Single group			Repeated group	
	HR ^b^ (95% CI)	*P* value		HR ^b^ (95% CI)	*P* value
Max SOFA	1.12 (1.05–1.20)	< 0.001		1.09 (1.03–1.16)	0.002
CCI	1.13 (1.04–1.21)	0.002		1.08 (1.00-1.15)	0.039
SAPS-II	1.02 (1.00-1.04)	0.038		1.03 (1.01–1.04)	< 0.001

^a^ Max SOFA, CCI and SAPS-II were included as continuous variables in subgroup analysis of Cox proportional hazard regressions

^b^ HRs were adjusted for some statistically significant factors according to multivariate analysis (Max SOFA, CCI, SAPS-II, respiratory failure, length of ICU stay and immunosuppressants/biologics)

Abbreviations: AD: autoimmune disease; CCI: Charlson comorbidity index; CI: confidence interval; HR: hazard ratio; SAPS-II: Simplified Acute Physiology Score-II; SOFA: Sequential Organ Failure Assessment

### The effect of risk factors on secondary outcomes

Table 4 and Supplementary Table S5 exhibited the effects of in-ICU sepsis frequency, Max SOFA, CCI and SAPS-II on two secondary outcomes (namely septic shock and in-ICU death). The risk of septic shock was found to be significantly higher in the repeated group compared to the single group (unadjusted odds ratio [OR] = 1.52, 95% CI: 1.05–2.23, *P* = 0.029; adjusted OR = 1.74, 95% CI: 1.15–2.66, *P* = 0.009). However, no association was observed between the repeated group and in-ICU death when compared to the single group (*P* > 0.05 both in univariate and multivariate logistical regression).

**Table 4:** Multivariate logistical regressions of secondary outcomes (septic shock and in-ICU death) among overall AD patients

Variables	Septic shock			In-ICU death	
	Adjusted OR ^a^ (95% CI)	*P* value		Adjusted OR ^a^ (95% CI)	*P* value
In-ICU sepsis frequency ^b^	1.74 (1.15–2.66)	0.009		1.39 (0.87–2.24)	0.174
Max SOFA ^c^	1.17 (1.10–1.25)	< 0.001		1.26 (1.18–1.36)	< 0.001
CCI ^c^	0.95 (0.87–1.03)	0.204		1.07 (0.98–1.18)	0.144
SAPS-II ^c^	1.02 (1.00-1.04)	0.043		1.03 (1.01–1.05)	0.008

^a^ ORs were adjusted for some potential variables of interest (in-ICUsepsis frequency, Max SOFA, CCI, SAPS-II, respiratory failure, length of ICU stay, and immunosuppressants/biologics)

^b^ In-ICU sepsis frequency acted as a dichotomous variable, and its ORs reflected the comparison between the repeated and the single group, with the single group as the reference

^c^ Max SOFA, CCI and SAPS-II were included as continuous variables

Abbreviations: AD: autoimmune disease; CCI: Charlson comorbidity index; CI: confidence interval; OR: odds ratio; ICU: intensive care unit; SAPS-II: Simplified Acute Physiology Score-II; SOFA: Sequential Organ Failure Assessment

For Max SOFA, CCI and SAPS-II among overall AD patients, increased scores of Max SOFA and SAPS-II correlated with septic shock and in-ICU death (all *P* < 0.05) except for CCI with insignificant differences in multivariate logistical regression (*P* > 0.05) (Table 4 and Supplementary Table S5). The effects of Max SOFA, CCI and SAPS-II on the secondary outcomes in the subgroups were shown in Supplementary Table S6.

## Discussion

To the best of our knowledge, this is the first retrospective cohort study focusing on the impact of repeated in-ICU sepsis on the survival of AD patients using the MIMIC database. We found that repeated in-ICU sepsis acted as a risk factor for 1-year overall-cause mortality and septic shock of AD patients. Furthermore, higher scores in Max SOFA, CCI and SAPS-II were also closely associated with poor prognosis among AD patients with sepsis.

Firstly, repeated in-ICU sepsis episode was significantly associated with higher 1-year overall-cause mortality among AD population, along with robust adjusted HRs in sensitivity analysis by the adjustment of confounders. The findings confirm that repeated in-ICU sepsis served as an independent risk factor for the long-term survival of AD individuals and its effect was less influenced by other covariates. Partly similar to our study, Pandolfi et al. [9] found that hospital readmission due to recurrent sepsis was associated with an increased risk of 1-year mortality. Likewise, some previous studies showed that readmission for recurrent sepsis was indeed a risk factor related to a higher mortality rate [18, 19]. Whereas, these studies on repeated sepsis rarely involved AD population. Other studies on ADs found that sepsis was an important factor influencing the mortality of ADs patients [5, 20], but they did not give a specific definition of whether the sepsis they studied was new-onset or recurrent. These studies provided some clues of the impact of sepsis on ADs, and our findings regarding the long-term prognosis of ADs in relation to recurrent sepsis helps made up for the limitations of these studies.

It remains unclear about the underlying pathogenesis of the increased vulnerability of AD with repeated sepsis to a poorer long-term prognosis; however, immune dysfunction may provide a potential explanation. Sepsis is a disorder as a result of the dysregulated host response to an infection, beginning with inflammation in the early phase and converting into an immunosuppression in the late phase [6, 7]. Previous studies showed that recurrent sepsis or post-sepsis state could induce the exhaustion and impaired response of CD4^+^ T cell and memory CD8^+^ T cell [21–23], along with the increase of regulatory T cells and myeloid derived suppressor cells that had T cell inhibiting capabilities [22, 24, 25]. Another study also identified HLA-DR^low^S100A^high^monocytes as a cell subtype enriched in the sepsis patients, which was also correlated to the immunosuppression [26]. In addition to the alteration of cell populations, immunosuppressive cytokine secretion and increased expression of negative costimulatory molecules were also found in the long-term immunosuppression status of sepsis [27]. The alteration of immunity might successively cause infection susceptibility and recurrent sepsis episodes. As such, the prolonged immunosuppression status and the recurrence of sepsis formed a bidirectional causality, perpetuating a detrimental loop and eventually causing organ dysfunctions even deaths. For AD populations in our study, the sepsis-induced immunosuppression might be also dysregulated and exacerbated by the ADs; in the meanwhile, the AD-associated multi-organ damage might coexist with the organ dysfunction induced by the sepsis. The underlying pathogenesis on the association between ADs and sepsis episodes needs more studies in the future.

Another important finding was that tools reflecting severe physical dysfunction were closely linked to AD patients with in-ICU sepsis. SOFA, CCI and SAPS-II, three commonly-used quantified assessment tools in ICU, were considered useful to assess organ dysfunction, disease severity, comorbidity status and prognosis of sepsis [12, 13, 28]. In our analysis of 1-year overall-cause mortality, we observed that higher scores in Max SOFA, CCI, and SAPS-II were risk factors for ADs patients. This association remained consistent even when conducting subgroup analyses where the repeated group showed a correlation between high scores of quantified assessment tools and mortality, resembling the findings observed within the single group (all *P* < 0.05). Moreover, within the repeated group, scores of the tools were higher during the last ICU stays in the comparison between the first admissions and the last ones (all *P* < 0.05), suggesting that sepsis developing repeatedly were more severely ill than those occurred at the first time. These findings are partly similar to other published literature reporting a correlation between Max SOFA, CCI, and SAPS-II scores and ADs [5, 29–31]. To summarize, the higher scores of three quantified assessment tools were long-term mortality-associated risk factors for AD patients whether with recurrent sepsis episodes or with single one. Those subjected to repeated sepsis had a propensity for higher scores after the previous hits of sepsis, indicating more severe physical dysfunction in the recurrence. As such, it is crucial to comprehensively assess the physical conditions and multiorgan involvements among AD patients with sepsis. Given a greater possibility of worse conditions, AD patients with repeated sepsis may require enhanced attention and long-term follow-up following a sepsis episode.

As for secondary outcomes, compared to those with single sepsis episode, individuals with repeated in-ICU sepsis had an increased risk of septic shock, while no significant differences were observed between the repeated and the single group on in-ICU death (or not) (Table 4). Nevertheless, the growth of Max SOFA scores and SAPS-II scores were correlated with both two secondary outcomes. The findings suggested that the septic shock was a vital outcome for AD patients with repeated sepsis. In terms of in-ICU death, one form of mortality in hospital, our patients had an ICU stay duration of one week or less (Table 1), suggesting in-ICU deaths were all within acute phases. During the acute phase, the occurrence of in-ICU death was associated with the severity of diseases (reflecting by SOFA or SAPS-II) instead of the frequency of sepsis episodes. In despite of the irrelevance, we reckon that the underlying mechanisms of in-ICU deaths were different between the single and the repeated group. For AD patients with once-only sepsis episode, early hyperinflammation might manifest in their initial hit, during which organ failure even deaths might happen due to uncontrolled inflammation as well as inadequate medical interventions [32–35]. On the other hand, for AD survivors with repeated sepsis, the prolonged immunosuppression made them lose the ability to resist the secondary infection and repeated sepsis, rendering them vulnerable to deaths during the acute phase [36, 37]. However, very few is still known about the association between in-ICU deaths and speiss episodes among AD population.

Respiratory failure, with acute ones occupying the majority (90.3% of overall respiratory failure before PSM), also emerged as another risk factor for long-term mortality, which was in line with previous study finding that respiratory failure was associated with the mortality of rheumatoid arthritis with sepsis [38]. On the other hand, the factors for a good prognosis in our study included longer length of ICU stay and the use of immunosuppressants. In the published literature, there were conflicting findings about the association between the length of ICU stay and mortality of AD patients. One study reported no significant relationship between the length of ICU stay and mortality of SLE patients [39], but it was restricted by small size of patient sample. Another study with a relatively large sample found that an extended ICU stay (2 ~ 14 days) was a risk factor for 3-year mortality for rheumatoid arthritis with sepsis [38], which was inconsistent with our results. But differently, the maximum ICU stay did not exceed 1 week in the baseline of our AD patients. The decreased risk of mortality observed among our AD patients who had a relatively longer ICU stay might be due to the supporting critical care and sufficient anti-sepsis therapy in an appropriate length of ICU stay [40]. As for immunosuppressants or biologics, a current study showed that the occurrence of sepsis among AD was not relevant to the use of immunosuppressants in the ICU [41]. but another small-scale study found biologics or corticosteroids could improve the 30-day survival of SLE patients with sepsis [39]. The contradiction of the results on ICU staying and drugs for ADs with sepsis needed more studies to resolve.

Our study had some strengths and limitations. A major strength was that our study was conducted based on a large patient sample and its design of a retrospective cohort study can better indicate the association between repeated sepsis and ADs. Also, variables of interest were adjusted by other confounders through post-PSM multivariate analysis and sensitivity analysis so as to make the results as reliable as possible. However, our study is subject to limitations imposed by the MIMIC database. Detailed causes of death were not available, which hindered further analysis of specific causes of mortality. In the meanwhile, due to privacy protection principles followed by the MIMIC database, the maximum follow-up period for each patient is limited to one year after the last discharge. Consequently, we were only able to focus on the 1-year overall-cause mortality as one of the outcomes. Secondly, we found it difficult to exclusively evaluate the disease activity and immunity of ADs due to the lack of some AD-specific measurements from the database, including autoantibody titer, cytokines, complements, the count of lymphocyte subtypes, etc. We could only utilize SOFA, CCI and SAPS-II, some quantified assessment tools commonly used in ICU, to assess the conditions of disease severity. As shown in our results, the tools indeed worked. Future studies should consider incorporating additional disease activity-related indices to further investigate the relationship between ADs and sepsis. Thirdly, this study utilized an open-access database derived from in-hospital database systems that were collected from routine clinical practice. It is possible that inaccuracies or non-standardization may exist in the documentations.

## Conclusion

This study demonstrated that repeated in-ICU sepsis was as a risk factor for 1-year overall-cause mortality among AD patients. Increased scores of Max SOFA, CCI and SAPS-II, which reflected severely ill physical conditions, were closely linked to unfavorable prognosis for AD patients experiencing repeated episodes. Thus, when AD patients encounter sepsis, physicians need more vigilance and pay more attention on their follow-up, especially among cases with multiple episodes. Future research endeavors and prospective trials are needed to unravel the underlying mechanisms.

### Electronic supplementary material

Below is the link to the electronic supplementary material.

Supplementary Material 1

## Data Availability

The datasets supporting the conclusions of this article are available in the Physionet (https://physionet.org/content/mimiciv/2.0/) [11].

## Abbreviations

AD: Autoimmune disease
CCI: Charlson comorbidity index
COPD: Chronic obstructive pulmonary disease
Hb: Hemoglobin
HIV: Human immunodeficiency virus
HR: Hazard ratio
ICU: Intensive care unit
IQR: Inter-quartile range
IVIG: Intravenous immunoglobulin
MIMIC-IV: Medical Information Mart for Intensive Care IV
OR: Odds ratio
PLT: Platelet
PSM: Propensity score matching
SAPS-II: Simplified Acute Physiology Score-II
SLE: Systemic lupus erythematosus
SOFA: Sequential Organ Failure Assessment
WBC: White blood cell
