# Supplementary material for: Prognostic impacts of repeated sepsis in intensive care unit on autoimmune disease patients: a retrospective cohort study
Source: BMC Infect Dis. 2024 Feb 13;24:197. doi: 10.1186/s12879-024-09072-y (PMC10863122; doi:10.1186/s12879-024-09072-y)
Supplement: Supplementary file 1 — Supplementary Material 1 [file 12879_2024_9072_MOESM1_ESM.docx]

**Supplemental materials**

Table S1 ICD-codes of autoimmune diseases and the number of each autoimmune disease patient

| Autoimmune disease | ICD-9 code | ICD-10 code | Number of patients |
| --- | --- | --- | --- |
| Systemic lupus erythematosus | 7100 | M32, M321, M3210, M3211, M3212, M3213, M3214, M3215, M3219, M328, M329 | 170 |
| Rheumatoid arthritis | 7140, 7142, 71481 | M05, M051, M0510, M0511, M05111, M05112, M05119, M0512, M05121, M05122, M05129, M0513, M05131, M05132, M05139, M0514, M05141, M05142, M05149, M0515, M05151, M05152, M05159, M0516, M05161, M05162, M05169, M0517, M05171, M05172, M05179, M0519, M052, M0520, M0521, M05211, M05212, M05219, M0522, M05221, M05222, M05229, M0523, M05231, M05232, M05239, M0524, M05241, M05242, M05249, M0525, M05251, M05252, M05259, M0526, M05261, M05262, M05269, M0527, M05271, M05272, M05279, M0529, M053, M0530, M0531, M05311, M05312, M05319, M0532, M05321, M05322, M05329, M0533, M05331, M05332, M05339, M0534, M05341, M05342, M05349, M0535, M05351, M05352, M05359, M0536, M05361, M05362, M05369, M0537, M05371, M05372, M05379, M0539, M054, M0540, M0541, M05411, M05412, M05419, M0542, M05421, M05422, M05429, M0543, M05431, M05432, M05439, M0544, M05441, M05442, M05449, M0545, M05451, M05452, M05459, M0546, M05461, M05462, M05469, M0547, M05471, M05472, M05479, M0549, M055, M0550, M0551, M05511, M05512, M05519, M0552, M05521, M05522, M05529, M0553, M05531, M05532, M05539, M0554, M05541, M05542, M05549, M0555, M05551, M05552, M05559, M0556, M05561, M05562, M05569, M0557, M05571, M05572, M05579, M0559, M056, M0560, M0561, M05611, M05612, M05619, M0562, M05621, M05622, M05629, M0563, M05631, M05632, M05639, M0564, M05641, M05642, M05649, M0565, M05651, M05652, M05659, M0566, M05661, M05662, M05669, M0567, M05671, M05672, M05679, M0569, M057, M0570, M0571, M05711, M05712, M05719, M0572, M05721, M05722, M05729, M0573, M05731, M05732, M05739, M0574, M05741, M05742, M05749, M0575, M05751, M05752, M05759, M0576, M05761, M05762, M05769, M0577, M05771, M05772, M05779, M0579, M058, M0580, M0581, M05811, M05812, M05819, M0582, M05821, M05822, M05829, M0583, M05831, M05832, M05839, M0584, M05841, M05842, M05849, M0585, M05851, M05852, M05859, M0586, M05861, M05862, M05869, M0587, M05871, M05872, M05879, M0589, M059, M06, M060, M0600, M0601, M06011, M06012, M06019, M0602, M06021, M06022, M06029, M0603, M06031, M06032, M06039, M0604, M06041, M06042, M06049, M0605, M06051, M06052, M06059, M0606, M06061, M06062, M06069, M0607, M06071, M06072, M06079, M0608, M0609, M068, M0680, M0681, M06811, M06812, M06819, M0682, M06821, M06822, M06829, M0683, M06831, M06832, M06839, M0684, M06841, M06842, M06849, M0685, M06851, M06852, M06859, M0686, M06861, M06862, M06869, M0687, M06871, M06872, M06879, M0688, M0689, M069 | 507 |
| Systemic sclerosis | 5172, 7101 | M34, M340, M341, M348, M3481, M3482, M3483, M3489, M349 | 51 |
| Psoriasis | 6960, 6961, 6968 | L40, L400, L401, L404, L405, L4050, L4051, L4052, L4053, L4059, L408, L409 | 341 |
| Ankylosing spondylitis | 7200 | M45, M450, M451, M452, M453, M454, M455, M456, M457, M458, M459, M46 | 43 |
| Vasculitis ^a^ | 1361, 4430, 4460, 4464, 4465, 4467, 71120, 71121, 71122, 71123, 71124, 71125, 71126, 71127, 71128, 71129 | M30, M300, M301, M303, M308, M313, M3130, M3131, M314, M315, M316, M317, M352 | 151 |
| Idiopathic inflammatory myopathies ^b^ | 7103, 7104, 35971, 35979 | G7241, M33, M331, M3310, M3311, M3312, M3313, M3319, M332, M3320, M3321, M3322, M3329, M339, M3390, M3391, M3392, M3393, M3399 | 30 |
| Crohn's disease | -- | K50, K500, K5000, K5001, K50011, K50012, K50013, K50014, K50018, K50019, K501, K5010, K5011, K50111, K50112, K50113, K50114, K50118, K50119, K508, K5080, K5081, K50811, K50812, K50813, K50814, K50818, K50819, K509, K5090, K5091, K50911, K50912, K50913, K50914, K50918, K50919 | 94 |
| Ulcerative colitis | 5560, 5561, 5562, 5563, 5565, 5566, 5568, 5569 | K51, K510, K5100, K5101, K51011, K51012, K51013, K51014, K51018, K51019, K512, K5120, K5121, K51211, K51212, K51213, K51214, K51218, K51219, K513, K5130, K5131, K51311, K51312, K51313, K51314, K51318, K51319, K518, K5180, K5181, K51811, K51812, K51813, K51814, K51818, K51819, K519, K5190, K5191, K51911, K51912, K51913, K51914, K51918, K51919 | 204 |
| Autoimmune hepatic diseases ^c^ | 57142 | K743, K754, K8301 | 96 |

^a^ Vasculitis included Behcet's syndrome, Raynaud's syndrome, polyarteritis nodosa, Wegener's granulomatosis, giant cell arteritis, Takayasu's disease, Churg-Strauss syndrome, Kawasaki's Disease and microscopic polyangiitis.

^b^ Idiopathic inflammatory myopathies included dermatomyositis, polymyositis and inclusion body myositis.

^c^ Autoimmune hepatic diseases included primary biliary cirrhosis, autoimmune hepatitis and primary sclerosing cholangitis.

Table S2 Category and medicines of drug therapies

| Category | Medicines |
| --- | --- |
| Glucocorticoids | Dexamethasone, methylprednisolone, hydrocortisone |
| Immunosuppressants/biologics | Azathioprine, chloroquine, hydroxychloroquine, leflunomide, cyclophosphamide, methotrexate, mycophenolate mofetil, tacrolimus, rituximab |

Table S3 Comparison of characteristics between the first admissions and the last admissions of ICU among the 315 AD patients with repeated in-ICU sepsis

| Variables | Total (n=315) | First admission | Last admission | *P* value |
| --- | --- | --- | --- | --- |
| Gender |  |  |  |  |
| Male | 128 (40.6%) |  |  |  |
| Female | 187 (59.4%) |  |  |  |
| Race |  |  |  |  |
| White | 219 (69.5%) |  |  |  |
| Black | 46 (14.6%) |  |  |  |
| Hispanic | 9 (2.9%) |  |  |  |
| Asian | 9 (2.9%) |  |  |  |
| Other | 14 (4.4%) |  |  |  |
| Unknown | 18 (5.7%) |  |  |  |
| Time interval of ICU admissions ^a^, days | 126.0 (15.0-604.5) |  |  |  |
| Length of ICU stay, days |  | 3 (1-6) | 3 (2-7) | 0.370 |
| **Comorbidities** |  |  |  |  |
| Acute kidney injury |  | 170 (54.0%) | 163 (51.7%) | 0.632 |
| Chronic kidney disease |  | 104 (33.0%) | 119 (37.8%) | 0.243 |
| Atrial fibrillation |  | 93 (29.5%) | 103 (32.7%) | 0.439 |
| Essential hypertension |  | 105 (33.3%) | 92 (29.2%) | 0.302 |
| Heart failure |  | 125 (39.7%) | 139 (44.1%) | 0.294 |
| Myocardial infarction |  | 48 (15.2%) | 57 (18.1%) | 0.392 |
| Respiratory failure |  | 130 (41.3%) | 142 (45.1%) | 0.376 |
| COPD |  | 20 (6.3%) | 29 (9.2%) | 0.234 |
| Diabetes mellitus |  | 112 (35.6%) | 122 (38.7%) | 0.458 |
| HIV infection |  | 3 (1.0%) | 3 (1.0%) | 1.000 |
| Malignancy |  | 26 (8.3%) | 25 (7.9%) | 1.000 |
| **Quantified assessment tools** | | | | |
| Max SOFA |  | 6 (4-9) | 6 (4-10) | 0.023 |
| CCI |  | 6 (5-8) | 7 (5-9) | 0.026 |
| SAPS-II |  | 37 (29-46) | 39 (31-48) | 0.013 |
| **Laboratory results** |  |  |  |  |
| Max WBC count, K/𝜇L |  | 15.50 (10.30-22.80) | 16.00 (10.70-23.00) | 0.646 |
| Min Hb, g/dL |  | 7.50 (6.70-8.80) | 7.20 (6.60-8.65) | 0.128 |
| Min PLT, K/𝜇L |  | 112.00 (58.00-180.00) | 119 (59.50-179.50) | 0.914 |
| **Drug therapies** | | | | |
| Glucocorticoid |  | 23 (7.3%) | 32 (10.2%) | 0.259 |
| Immunosuppressants/biologics |  | 58 (18.4%) | 46 (14.6%) | 0.238 |
| IVIG |  | 14 (4.4%) | 14 (4.4%) | 1.000 |

^a^ Time interval of ICU admissions was defined as the date of the last ICU admission minus that of ICU discharge in the first admission.

Abbreviations: AD, autoimmune disease; CCI, Charlson comorbidity index; COPD, chronic obstructive pulmonary disease; Hb, hemoglobin; HIV, human immunodeficiency virus; ICU, intensive care unit; IVIG, intravenous immunoglobulin; PLT, platelet; PSM, propensity score matching; SAPS-II, Simplified Acute Physiology Score-II; SOFA, Sequential Organ Failure Assessment; WBC, white blood cell.

Table S4 Sensitivity analysis of in-ICU sepsis frequency on one-year overall-cause mortality among overall AD patients

|  | HR ^a^ | 95% CI | *P* value |
| --- | --- | --- | --- |
| Unadjusted model ^b^ | 1.32 | 1.03-1.70 | 0.028 |
| Model 1 ^c^ | 1.41 | 1.10-1.81 | 0.007 |
| Model 2 ^d^ | 1.45 | 1.13-1.87 | 0.004 |
| Model 3 ^e^ | 1.49 | 1.16-1.92 | 0.002 |

^a^ The HRs reflected in-ICU sepsis frequency, and other variables included in Model 1-3 set as covariates

^b^ Unadjusted models was equal to univariate Cox analysis.

^c^ Model 1: hazard ratio (HR) was adjusted for in-ICU sepsis frequency, Max SOFA, CCI and SAPS-II.

^d^ Model 2: HR was adjusted for in-ICU sepsis frequency, Max SOFA, CCI, SAPS-II and respiratory failure.

^e^ Model 3: HR was adjusted for all statistically signiﬁcant factors according to multivariate analysis (including in-ICU sepsis frequency, Max SOFA, CCI, SAPS-II, respiratory failure, length of ICU stay and immunosuppressants/biologics).

Abbreviations: AD, autoimmune disease; CCI, Charlson comorbidity index; CI, confidence interval; HR, hazard ratio; ICU, intensive care unit; SAPS-II, Simplified Acute Physiology Score-II; SOFA, Sequential Organ Failure Assessment.

Table S5 Univariate logistical regressions of secondary outcomes (septic shock and in-ICU death) among overall AD patients

| Variables | Septic shock | |  |  | In-ICU death | | |
| --- | --- | --- | --- | --- | --- | --- | --- |
|  | Unadjusted OR (95% CI) | *P* value | Unadjusted OR (95% CI) | | | *P* value |  |
| In-ICU sepsis frequency ^a^ | 1.52 (1.05-2.23) | 0.029 | 1.15 (0.78-1.71) | | | 0.482 |  |
| Max SOFA ^b^ | 1.21 (1.15-1.27) | <0.001 | 1.32 (1.25-1.40) | | | <0.001 |  |
| CCI ^b^ | 1.03 (0.96-1.10) | 0.442 | 1.14 (1.06-1.22) | | | <0.001 |  |
| SAPS-II ^b^ | 1.04 (1.03-1.06) | <0.001 | 1.07 (1.05-1.08) | | | <0.001 |  |

^a^ In-ICU sepsis frequency acted as a dichotomous variable, and its ORs reflected the comparison between the repeated and the single group (reference = the single group).

^b^ Max SOFA, CCI and SAPS-II were included as continuous variables.

Abbreviations: AD, autoimmune disease; CCI, Charlson comorbidity index; CI, confidence interval; OR, odds ratio; ICU, intensive care unit; SAPS-II, Simplified Acute Physiology Score-II; SOFA, Sequential Organ Failure Assessment.

Table S6 Subgroup analysis of quantified assessment tool on secondary outcomes (septic shock and in-ICU death) among the single and the repeated group

| Variables | Septic shock | |  | In-ICU death | |
| --- | --- | --- | --- | --- | --- |
|  | Adjusted OR ^a^ (95% CI) | *P* value |  | Adjusted OR ^a^ (95% CI) | *P* value |
| **Single group** | | | | | |
| Max SOFA ^b^ | 1.09 (0.99-1.21) | 0.072 |  | 1.24 (1.12-1.39) | <0.001 |
| CCI ^b^ | 0.98 (0.86-1.11) | 0.742 |  | 1.11 (0.97-1.28) | 0.128 |
| SAPS-II ^b^ | 1.03 (1.01-1.06) | 0.021 |  | 1.02 (0.99-1.05) | 0.193 |
| **Repeated group** | | | | | |
| Max SOFA ^b^ | 1.26 (1.15-1.39) | <0.001 |  | 1.28 (1.16-1.43) | <0.001 |
| CCI ^b^ | 0.91 (0.80-1.02) | 0.101 |  | 1.04 (0.90-1.19) | 0.596 |
| SAPS-II ^b^ | 1.01 (0.98-1.03) | 0.641 |  | 1.04 (1.01-1.07) | 0.014 |

^a^ ORs were adjusted for some potential variables of interest (Max SOFA, CCI, SAPS-II, respiratory failure, length of ICU stay and immunosuppressants/biologics).

^b^ Max SOFA, CCI and SAPS-II were included as continuous variables.

Abbreviations: AD, autoimmune disease; CCI, Charlson comorbidity index; CI, confidence interval; OR, odds ratio; ICU, intensive care unit; SAPS-II, Simplified Acute Physiology Score-II; SOFA, Sequential Organ Failure Assessment.
